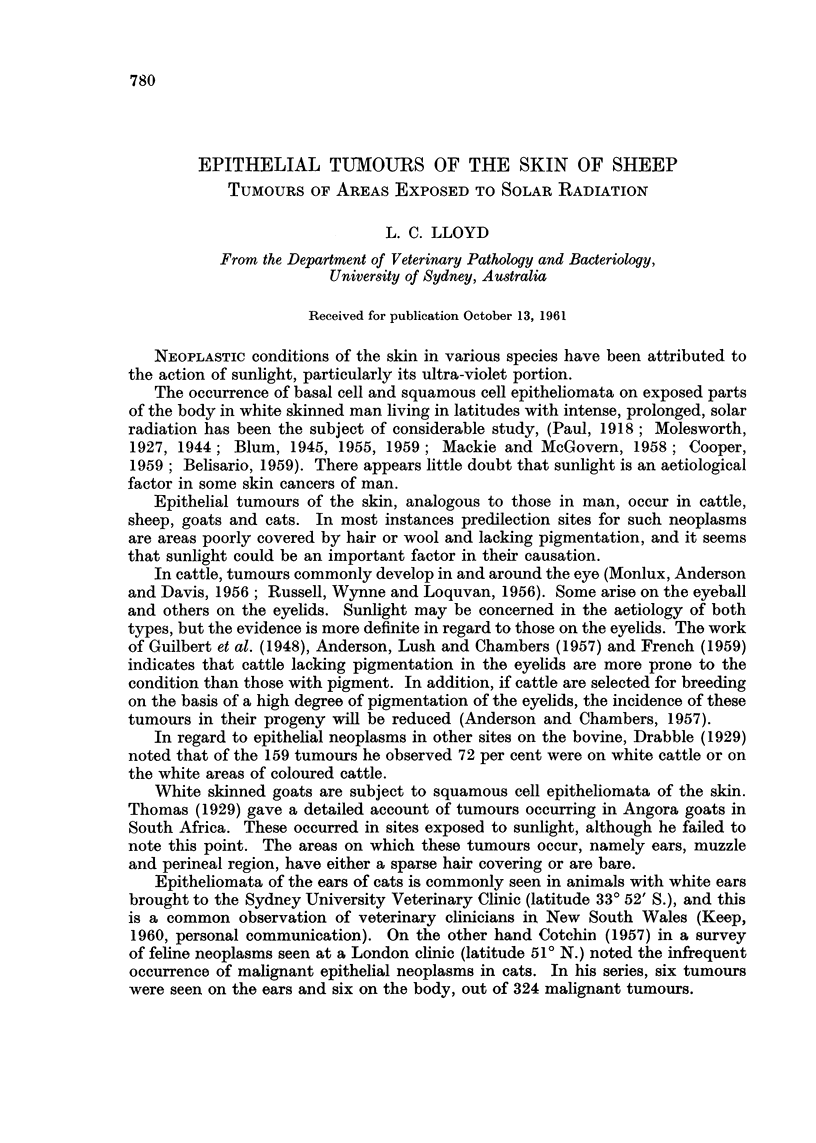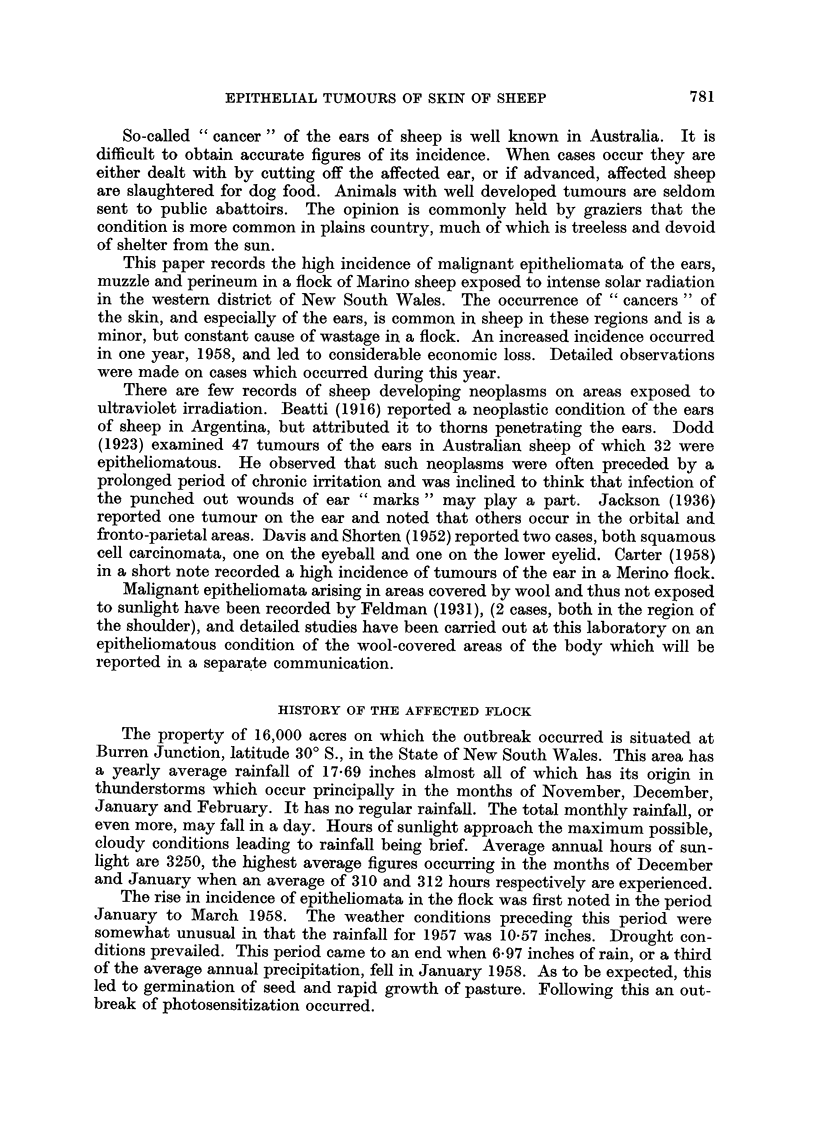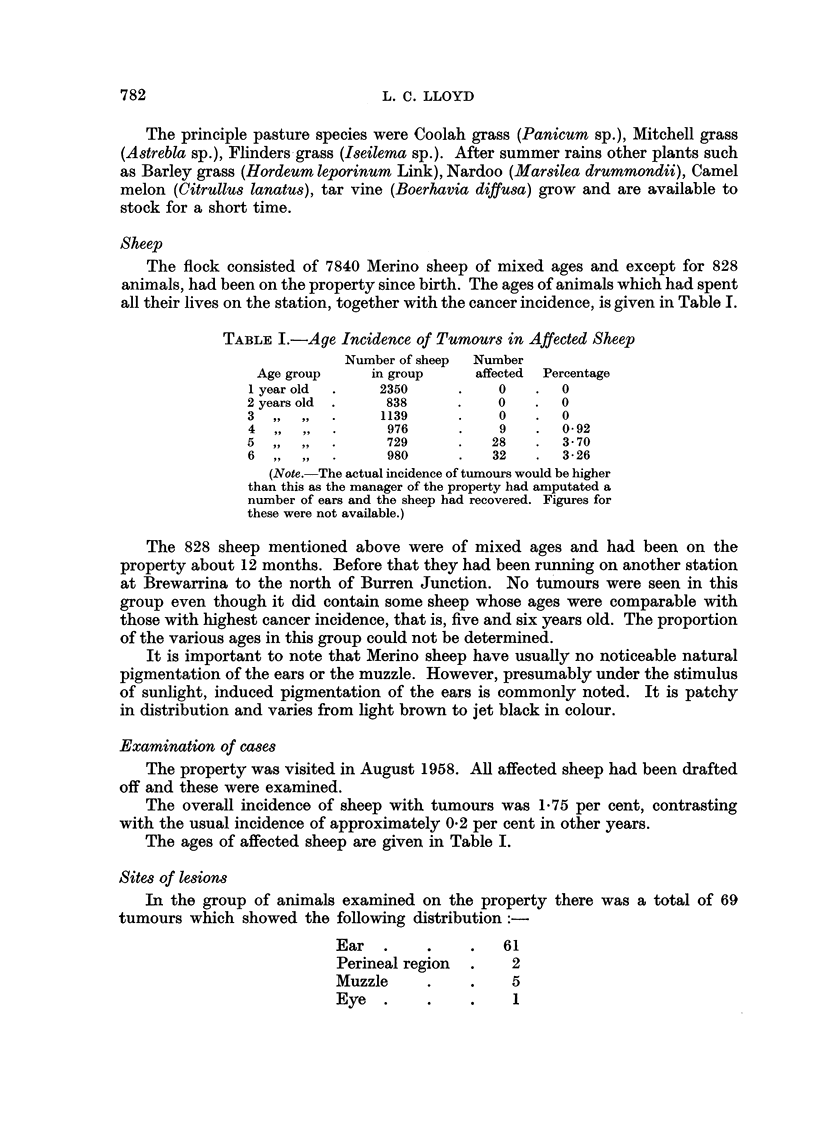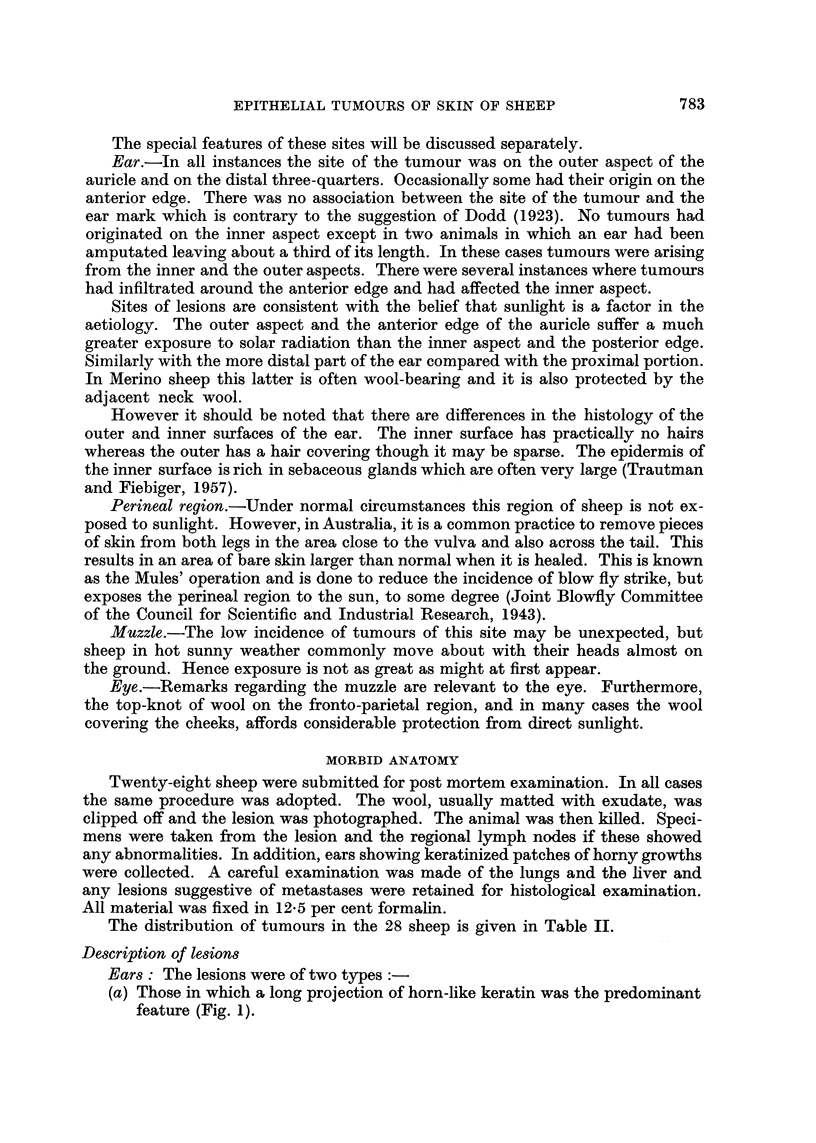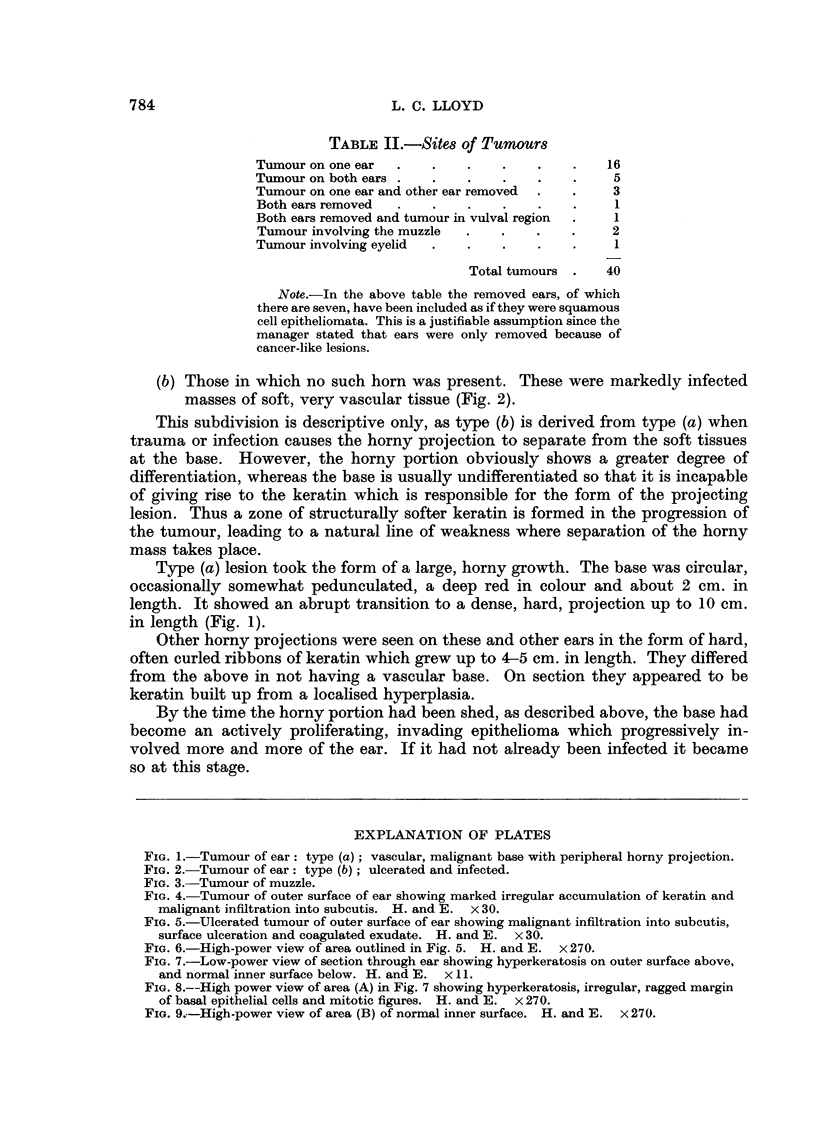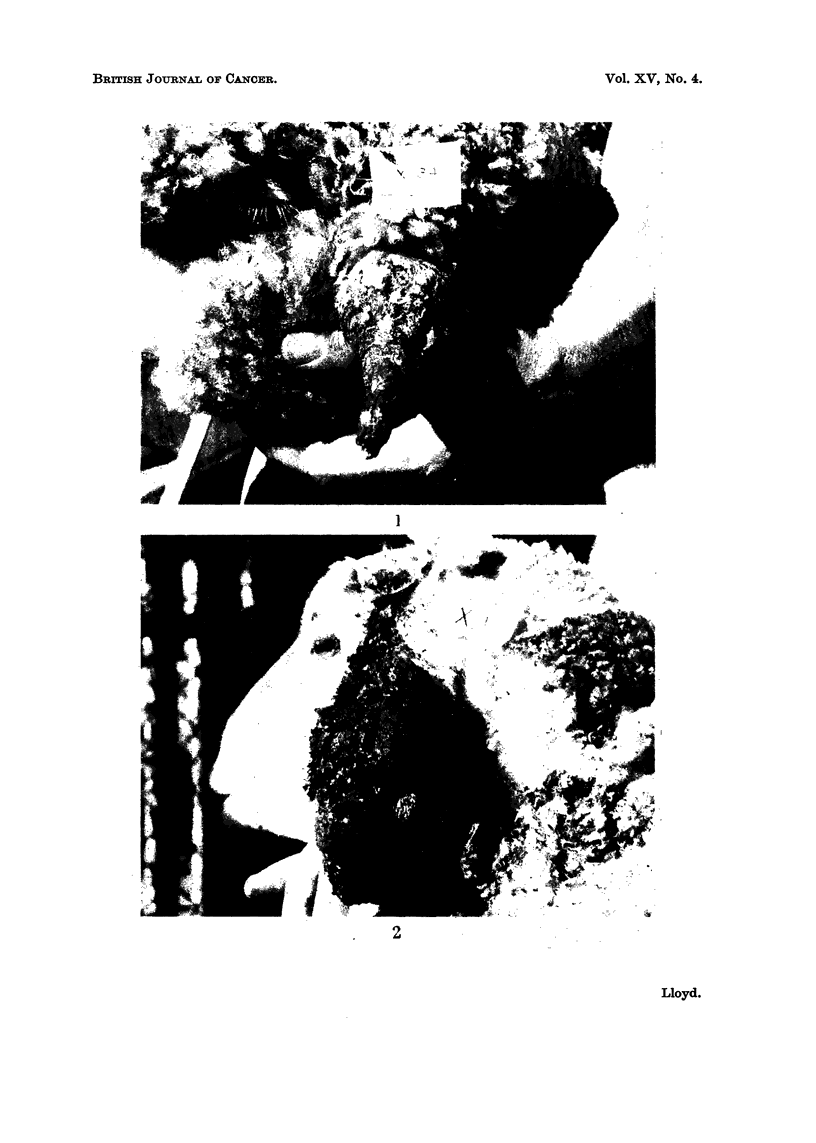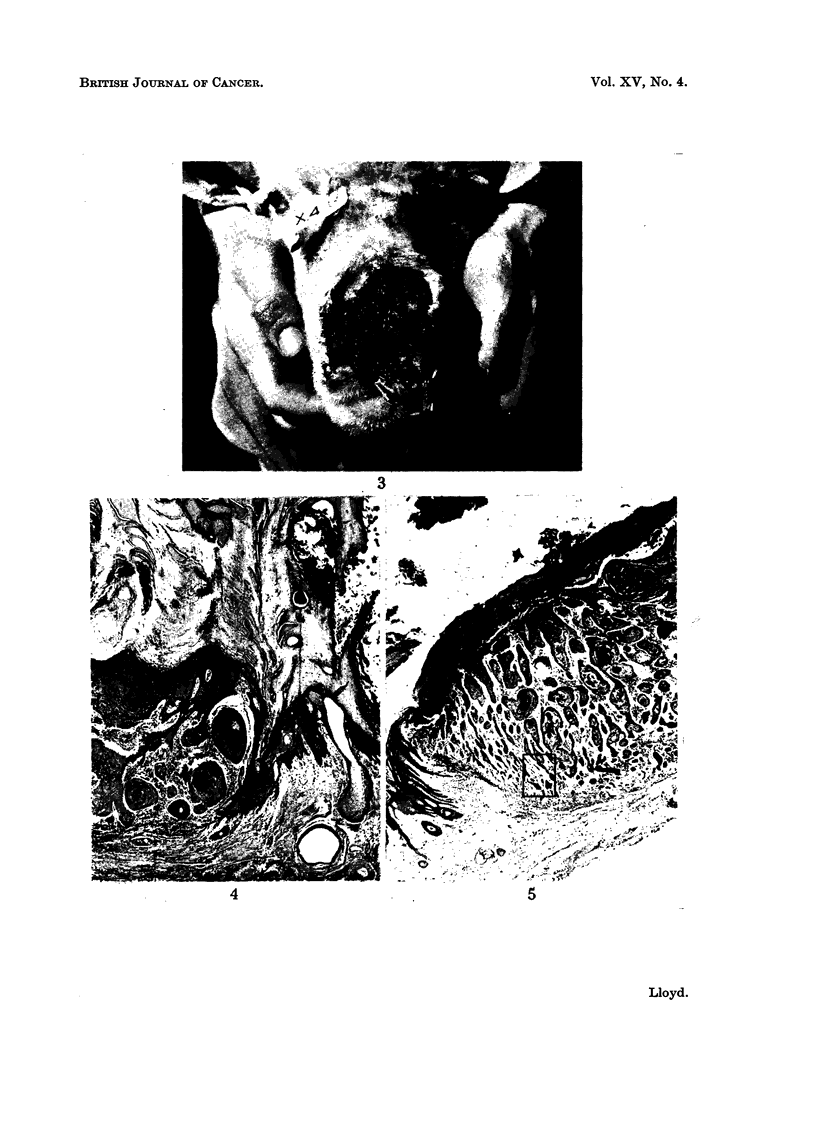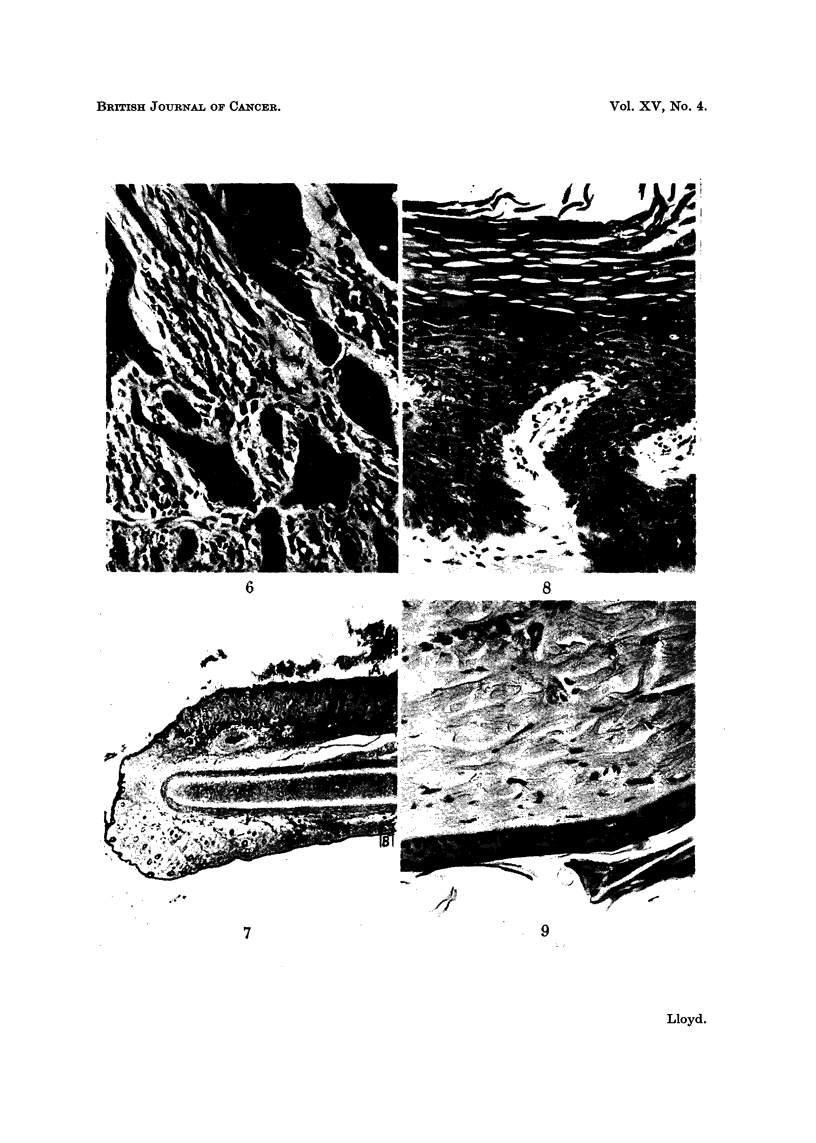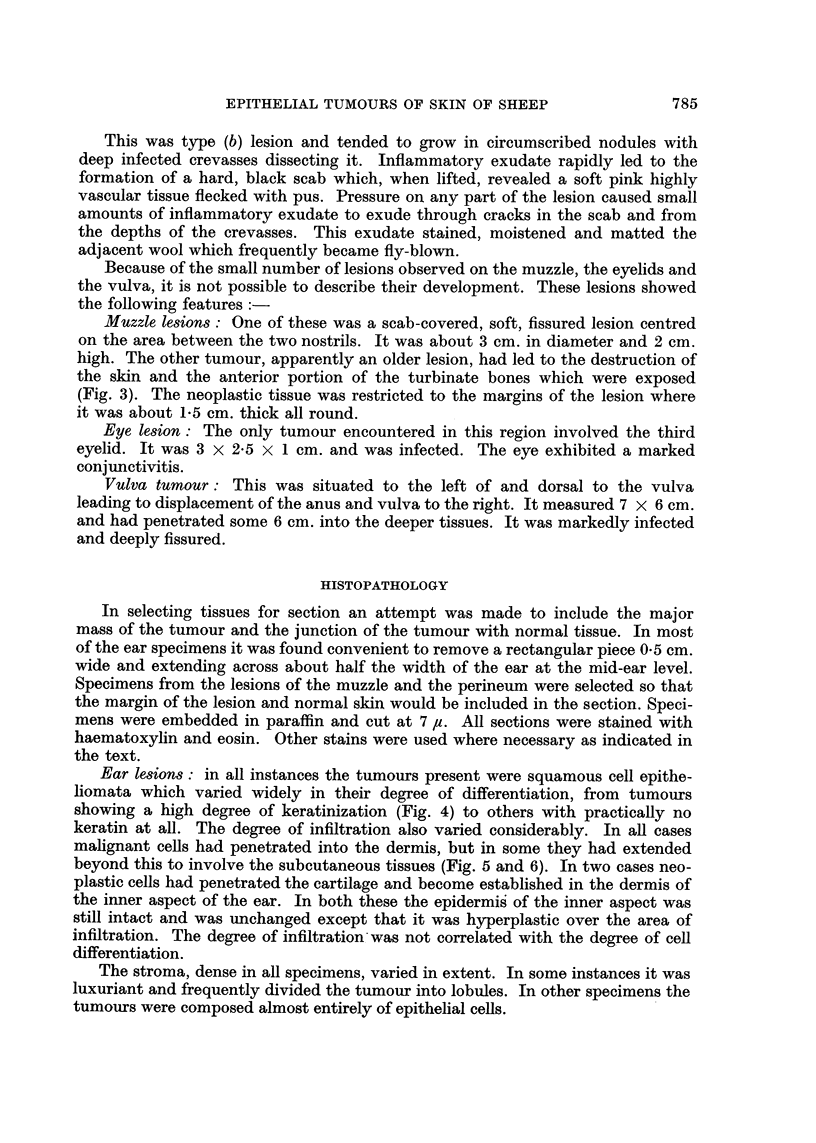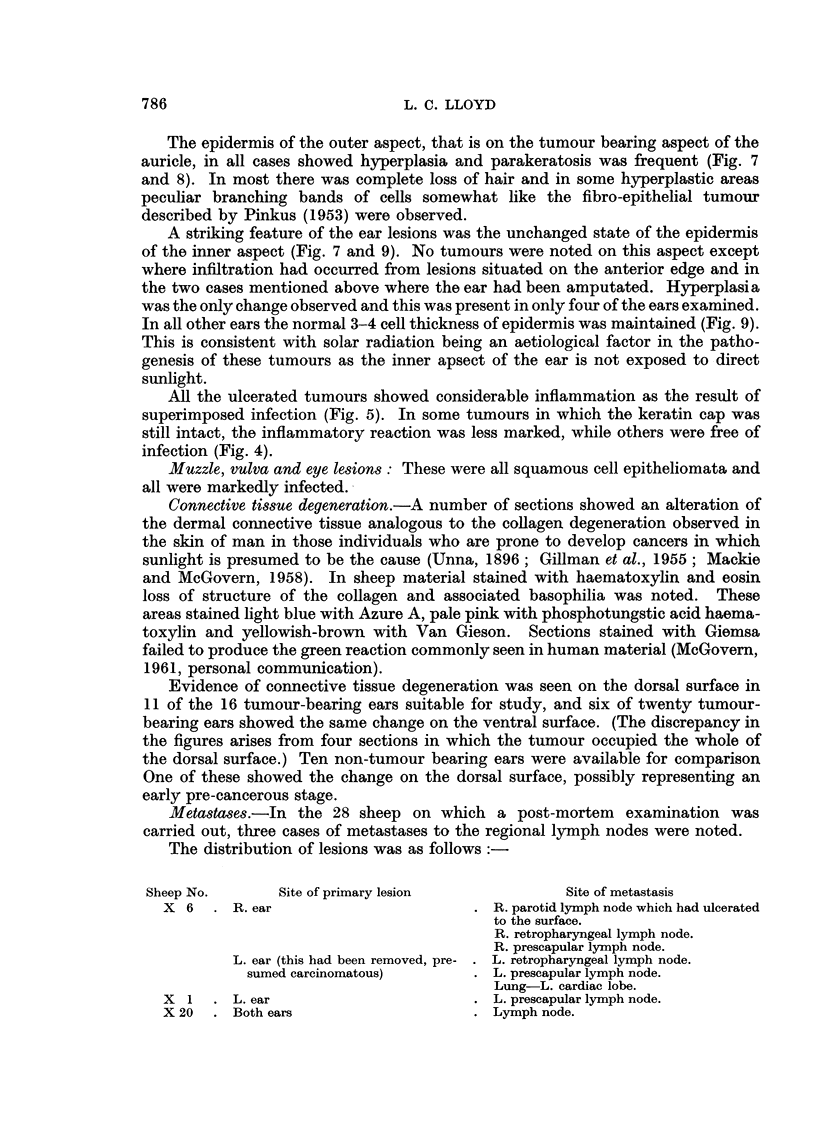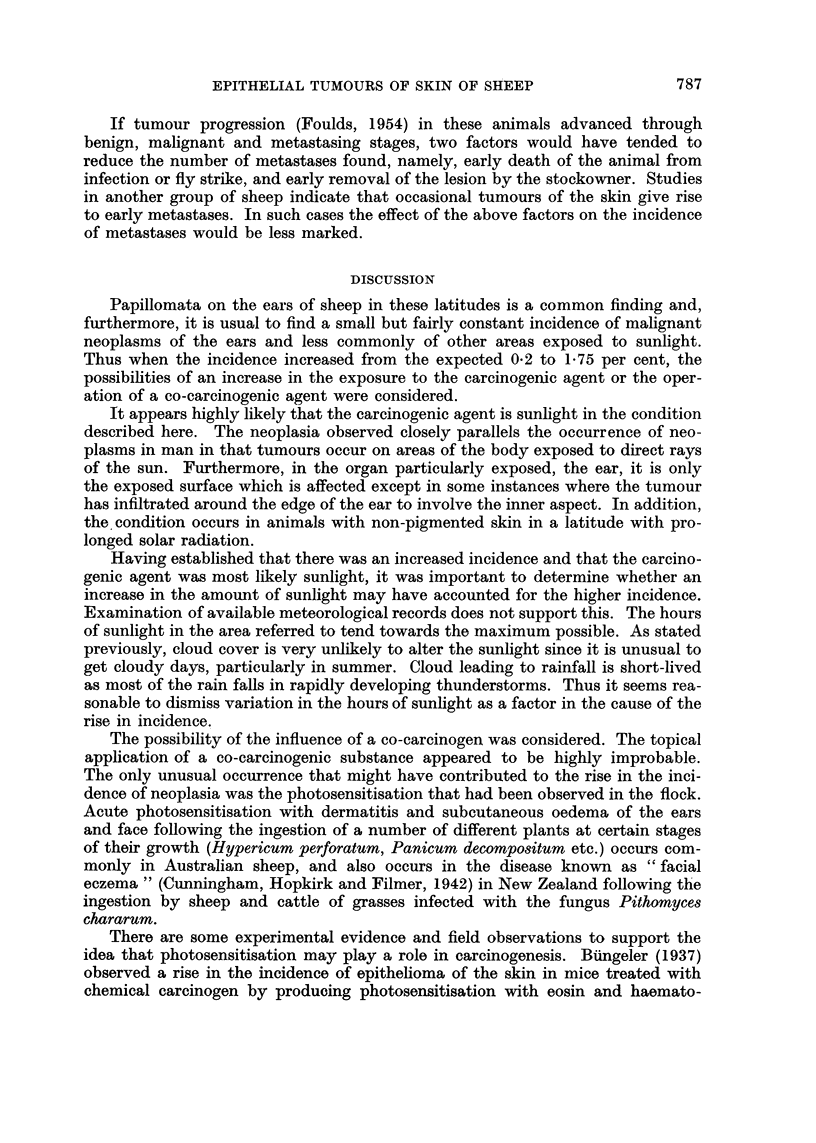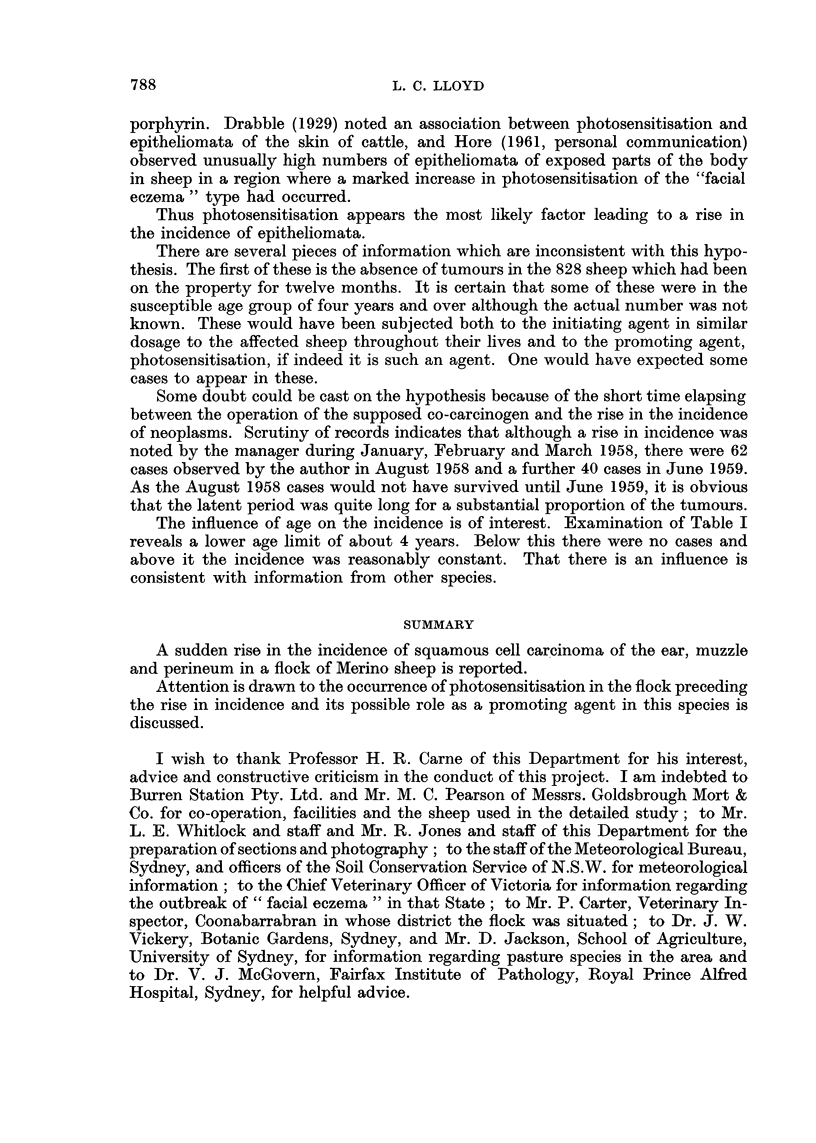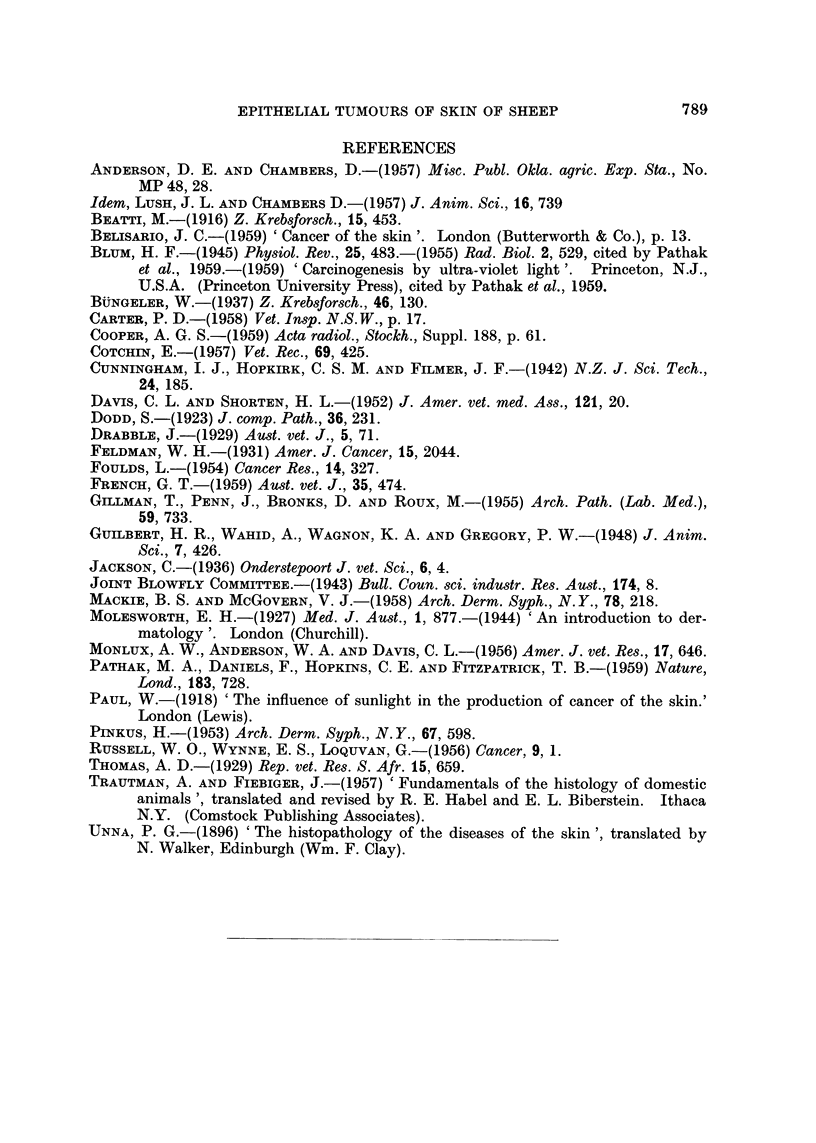# Epithelial Tumours of the Skin of Sheep

**DOI:** 10.1038/bjc.1961.88

**Published:** 1961-12

**Authors:** L. C. Lloyd

## Abstract

**Images:**


### EPITHELIAL TUMOURS OF THE SKIN OF SHEEP
### Tumours of Areas Exposed to Solar Radiation

L. C. LLOYD

*From the Department of Veterinary Pathology and Bacteriology, University of Sydney, Australia*

Received for publication October 13, 1961

Neoplastic conditions of the skin in various species have been attributed to the action of sunlight, particularly its ultra-violet portion.

The occurrence of basal cell and squamous cell epitheliomata on exposed parts of the body in white skinned man living in latitudes with intense, prolonged, solar radiation has been the subject of considerable study, (Paul, 1918 ; Molesworth, 1927, 1944 ; Blum, 1945, 1955, 1959 ; Mackie and McGovern, 1958 ; Cooper, 1959 ; Belisario, 1959). There appears little doubt that sunlight is an aetiological factor in some skin cancers of man.

Epithelial tumours of the skin, analogous to those in man, occur in cattle, sheep, goats and cats. In most instances predilection sites for such neoplasms are areas poorly covered by hair or wool and lacking pigmentation, and it seems that sunlight could be an important factor in their causation.

In cattle, tumours commonly develop in and around the eye (Monlux, Anderson and Davis, 1956 ; Russell, Wynne and Loquvan, 1956). Some arise on the eyeball and others on the eyelids. Sunlight may be concerned in the aetiology of both types, but the evidence is more definite in regard to those on the eyelids. The work of Guilbert *et al.* (1948), Anderson, Lush and Chambers (1957) and French (1959) indicates that cattle lacking pigmentation in the eyelids are more prone to the condition than those with pigment. In addition, if cattle are selected for breeding on the basis of a high degree of pigmentation of the eyelids, the incidence of these tumours in their progeny will be reduced (Anderson and Chambers, 1957).

In regard to epithelial neoplasms in other sites on the bovine, Drabble (1929) noted that of the 159 tumours he observed 72 per cent were on white cattle or on the white areas of coloured cattle.

White skinned goats are subject to squamous cell epitheliomata of the skin. Thomas (1929) gave a detailed account of tumours occurring in Angora goats in South Africa. These occurred in sites exposed to sunlight, although he failed to note this point. The areas on which these tumours occur, namely ears, muzzle and perineal region, have either a sparse hair covering or are bare.

Epitheliomata of the ears of cats is commonly seen in animals with white ears brought to the Sydney University Veterinary Clinic (latitude 33° 52′ S.), and this is a common observation of veterinary clinicians in New South Wales (Keep, 1960, personal communication). On the other hand Cotchin (1957) in a survey of feline neoplasms seen at a London clinic (latitude 51° N.) noted the infrequent occurrence of malignant epithelial neoplasms in cats. In his series, six tumours were seen on the ears and six on the body, out of 324 malignant tumours.

So-called " cancer " of the ears of sheep is well known in Australia. It is difficult to obtain accurate figures of its incidence. When cases occur they are either dealt with by cutting off the affected ear, or if advanced, affected sheep are slaughtered for dog food. Animals with well developed tumours are seldom sent to public abattoirs. The opinion is commonly held by graziers that the condition is more common in plains country, much of which is treeless and devoid of shelter from the sun.

This paper records the high incidence of malignant epitheliomata of the ears, muzzle and perineum in a flock of Marino sheep exposed to intense solar radiation in the western district of New South Wales. The occurrence of " cancers " of the skin, and especially of the ears, is common in sheep in these regions and is a minor, but constant cause of wastage in a flock. An increased incidence occurred in one year, 1958, and led to considerable economic loss. Detailed observations were made on cases which occurred during this year.

There are few records of sheep developing neoplasms on areas exposed to ultraviolet irradiation. Beatti (1916) reported a neoplastic condition of the ears of sheep in Argentina, but attributed it to thorns penetrating the ears. Dodd (1923) examined 47 tumours of the ears in Australian sheep of which 32 were epitheliomatous. He observed that such neoplasms were often preceded by a prolonged period of chronic irritation and was inclined to think that infection of the punched out wounds of ear " marks " may play a part. Jackson (1936) reported one tumour on the ear and noted that others occur in the orbital and fronto-parietal areas. Davis and Shorten (1952) reported two cases, both squamous cell carcinomata, one on the eyeball and one on the lower eyelid. Carter (1958) in a short note recorded a high incidence of tumours of the ear in a Merino flock.

Malignant epitheliomata arising in areas covered by wool and thus not exposed to sunlight have been recorded by Feldman (1931), (2 cases, both in the region of the shoulder), and detailed studies have been carried out at this laboratory on an epitheliomatous condition of the wool-covered areas of the body which will be reported in a separate communication.

### HISTORY OF THE AFFECTED FLOCK

The property of 16,000 acres on which the outbreak occurred is situated at Burren Junction, latitude 30° S., in the State of New South Wales. This area has a yearly average rainfall of 17·69 inches almost all of which has its origin in thunderstorms which occur principally in the months of November, December, January and February. It has no regular rainfall. The total monthly rainfall, or even more, may fall in a day. Hours of sunlight approach the maximum possible, cloudy conditions leading to rainfall being brief. Average annual hours of sunlight are 3250, the highest average figures occurring in the months of December and January when an average of 310 and 312 hours respectively are experienced.

The rise in incidence of epitheliomata in the flock was first noted in the period January to March 1958. The weather conditions preceding this period were somewhat unusual in that the rainfall for 1957 was 10·57 inches. Drought conditions prevailed. This period came to an end when 6·97 inches of rain, or a third of the average annual precipitation, fell in January 1958. As to be expected, this led to germination of seed and rapid growth of pasture. Following this an outbreak of photosensitization occurred.

The principle pasture species were Coolah grass (*Panicum* sp.), Mitchell grass (*Astrebla* sp.), Flinders grass (*Iseilema* sp.). After summer rains other plants such as Barley grass (*Hordeum leporinum* Link), Nardoo (*Marsilea drummondii*), Camel melon (*Citrullus lanatus*), tar vine (*Boerhavia diffusa*) grow and are available to stock for a short time.

### *Sheep*

The flock consisted of 7840 Merino sheep of mixed ages and except for 828 animals, had been on the property since birth. The ages of animals which had spent all their lives on the station, together with the cancer incidence, is given in Table I.

TABLE I.—*Age Incidence of Tumours in Affected Sheep*

| Age group | Number of sheep in group | Number affected | Percentage |
|---|---|---|---|
| 1 year old | 2350 | 0 | 0 |
| 2 years old | 838 | 0 | 0 |
| 3 ,, ,, | 1139 | 0 | 0 |
| 4 ,, ,, | 976 | 9 | 0·92 |
| 5 ,, ,, | 729 | 28 | 3·70 |
| 6 ,, ,, | 980 | 32 | 3·26 |

(*Note.*—The actual incidence of tumours would be higher than this as the manager of the property had amputated a number of ears and the sheep had recovered. Figures for these were not available.)

The 828 sheep mentioned above were of mixed ages and had been on the property about 12 months. Before that they had been running on another station at Brewarrina to the north of Burren Junction. No tumours were seen in this group even though it did contain some sheep whose ages were comparable with those with highest cancer incidence, that is, five and six years old. The proportion of the various ages in this group could not be determined.

It is important to note that Merino sheep have usually no noticeable natural pigmentation of the ears or the muzzle. However, presumably under the stimulus of sunlight, induced pigmentation of the ears is commonly noted. It is patchy in distribution and varies from light brown to jet black in colour.

### *Examination of cases*

The property was visited in August 1958. All affected sheep had been drafted off and these were examined.

The overall incidence of sheep with tumours was 1·75 per cent, contrasting with the usual incidence of approximately 0·2 per cent in other years.

The ages of affected sheep are given in Table I.

### *Sites of lesions*

In the group of animals examined on the property there was a total of 69 tumours which showed the following distribution :—

| | |
|---|---|
| Ear | 61 |
| Perineal region | 2 |
| Muzzle | 5 |
| Eye | 1 |

The special features of these sites will be discussed separately.

*Ear.*—In all instances the site of the tumour was on the outer aspect of the auricle and on the distal three-quarters. Occasionally some had their origin on the anterior edge. There was no association between the site of the tumour and the ear mark which is contrary to the suggestion of Dodd (1923). No tumours had originated on the inner aspect except in two animals in which an ear had been amputated leaving about a third of its length. In these cases tumours were arising from the inner and the outer aspects. There were several instances where tumours had infiltrated around the anterior edge and had affected the inner aspect.

Sites of lesions are consistent with the belief that sunlight is a factor in the aetiology. The outer aspect and the anterior edge of the auricle suffer a much greater exposure to solar radiation than the inner aspect and the posterior edge. Similarly with the more distal part of the ear compared with the proximal portion. In Merino sheep this latter is often wool-bearing and it is also protected by the adjacent neck wool.

However it should be noted that there are differences in the histology of the outer and inner surfaces of the ear. The inner surface has practically no hairs whereas the outer has a hair covering though it may be sparse. The epidermis of the inner surface is rich in sebaceous glands which are often very large (Trautman and Fiebiger, 1957).

*Perineal region.*—Under normal circumstances this region of sheep is not exposed to sunlight. However, in Australia, it is a common practice to remove pieces of skin from both legs in the area close to the vulva and also across the tail. This results in an area of bare skin larger than normal when it is healed. This is known as the Mules' operation and is done to reduce the incidence of blow fly strike, but exposes the perineal region to the sun, to some degree (Joint Blowfly Committee of the Council for Scientific and Industrial Research, 1943).

*Muzzle.*—The low incidence of tumours of this site may be unexpected, but sheep in hot sunny weather commonly move about with their heads almost on the ground. Hence exposure is not as great as might at first appear.

*Eye.*—Remarks regarding the muzzle are relevant to the eye. Furthermore, the top-knot of wool on the fronto-parietal region, and in many cases the wool covering the cheeks, affords considerable protection from direct sunlight.

### MORBID ANATOMY

Twenty-eight sheep were submitted for post mortem examination. In all cases the same procedure was adopted. The wool, usually matted with exudate, was clipped off and the lesion was photographed. The animal was then killed. Specimens were taken from the lesion and the regional lymph nodes if these showed any abnormalities. In addition, ears showing keratinized patches of horny growths were collected. A careful examination was made of the lungs and the liver and any lesions suggestive of metastases were retained for histological examination. All material was fixed in 12·5 per cent formalin.

The distribution of tumours in the 28 sheep is given in Table II.

### *Description of lesions*

*Ears :* The lesions were of two types :—

(*a*) Those in which a long projection of horn-like keratin was the predominant feature (Fig. 1).

TABLE II.—*Sites of Tumours*

| | |
|---|---|
| Tumour on one ear | 16 |
| Tumour on both ears | 5 |
| Tumour on one ear and other ear removed | 3 |
| Both ears removed | 1 |
| Both ears removed and tumour in vulval region | 1 |
| Tumour involving the muzzle | 2 |
| Tumour involving eyelid | 1 |
| Total tumours | 40 |

*Note.*—In the above table the removed ears, of which there are seven, have been included as if they were squamous cell epitheliomata. This is a justifiable assumption since the manager stated that ears were only removed because of cancer-like lesions.

(*b*) Those in which no such horn was present. These were markedly infected masses of soft, very vascular tissue (Fig. 2).

This subdivision is descriptive only, as type (*b*) is derived from type (*a*) when trauma or infection causes the horny projection to separate from the soft tissues at the base. However, the horny portion obviously shows a greater degree of differentiation, whereas the base is usually undifferentiated so that it is incapable of giving rise to the keratin which is responsible for the form of the projecting lesion. Thus a zone of structurally softer keratin is formed in the progression of the tumour, leading to a natural line of weakness where separation of the horny mass takes place.

Type (*a*) lesion took the form of a large, horny growth. The base was circular, occasionally somewhat pedunculated, a deep red in colour and about 2 cm. in length. It showed an abrupt transition to a dense, hard, projection up to 10 cm. in length (Fig. 1).

Other horny projections were seen on these and other ears in the form of hard, often curled ribbons of keratin which grew up to 4–5 cm. in length. They differed from the above in not having a vascular base. On section they appeared to be keratin built up from a localised hyperplasia.

By the time the horny portion had been shed, as described above, the base had become an actively proliferating, invading epithelioma which progressively involved more and more of the ear. If it had not already been infected it became so at this stage.

---

EXPLANATION OF PLATES

FIG. 1.—Tumour of ear: type (*a*); vascular, malignant base with peripheral horny projection.
FIG. 2.—Tumour of ear: type (*b*); ulcerated and infected.
FIG. 3.—Tumour of muzzle.
FIG. 4.—Tumour of outer surface of ear showing marked irregular accumulation of keratin and malignant infiltration into subcutis. H. and E. ×30.
FIG. 5.—Ulcerated tumour of outer surface of ear showing malignant infiltration into subcutis, surface ulceration and coagulated exudate. H. and E. ×30.
FIG. 6.—High-power view of area outlined in Fig. 5. H. and E. ×270.
FIG. 7.—Low-power view of section through ear showing hyperkeratosis on outer surface above, and normal inner surface below. H. and E. ×11.
FIG. 8.—High power view of area (A) in Fig. 7 showing hyperkeratosis, irregular, ragged margin of basal epithelial cells and mitotic figures. H. and E. ×270.
FIG. 9.—High-power view of area (B) of normal inner surface. H. and E. ×270.

1

2

Lloyd.

3

4

5

6

8

7

9

This was type (*b*) lesion and tended to grow in circumscribed nodules with deep infected crevasses dissecting it. Inflammatory exudate rapidly led to the formation of a hard, black scab which, when lifted, revealed a soft pink highly vascular tissue flecked with pus. Pressure on any part of the lesion caused small amounts of inflammatory exudate to exude through cracks in the scab and from the depths of the crevasses. This exudate stained, moistened and matted the adjacent wool which frequently became fly-blown.

Because of the small number of lesions observed on the muzzle, the eyelids and the vulva, it is not possible to describe their development. These lesions showed the following features :—

*Muzzle lesions :* One of these was a scab-covered, soft, fissured lesion centred on the area between the two nostrils. It was about 3 cm. in diameter and 2 cm. high. The other tumour, apparently an older lesion, had led to the destruction of the skin and the anterior portion of the turbinate bones which were exposed (Fig. 3). The neoplastic tissue was restricted to the margins of the lesion where it was about 1·5 cm. thick all round.

*Eye lesion :* The only tumour encountered in this region involved the third eyelid. It was 3 × 2·5 × 1 cm. and was infected. The eye exhibited a marked conjunctivitis.

*Vulva tumour :* This was situated to the left of and dorsal to the vulva leading to displacement of the anus and vulva to the right. It measured 7 × 6 cm. and had penetrated some 6 cm. into the deeper tissues. It was markedly infected and deeply fissured.

### HISTOPATHOLOGY

In selecting tissues for section an attempt was made to include the major mass of the tumour and the junction of the tumour with normal tissue. In most of the ear specimens it was found convenient to remove a rectangular piece 0·5 cm. wide and extending across about half the width of the ear at the mid-ear level. Specimens from the lesions of the muzzle and the perineum were selected so that the margin of the lesion and normal skin would be included in the section. Specimens were embedded in paraffin and cut at 7 $\mu$. All sections were stained with haematoxylin and eosin. Other stains were used where necessary as indicated in the text.

*Ear lesions :* in all instances the tumours present were squamous cell epitheliomata which varied widely in their degree of differentiation, from tumours showing a high degree of keratinization (Fig. 4) to others with practically no keratin at all. The degree of infiltration also varied considerably. In all cases malignant cells had penetrated into the dermis, but in some they had extended beyond this to involve the subcutaneous tissues (Fig. 5 and 6). In two cases neoplastic cells had penetrated the cartilage and become established in the dermis of the inner aspect of the ear. In both these the epidermis of the inner aspect was still intact and was unchanged except that it was hyperplastic over the area of infiltration. The degree of infiltration was not correlated with the degree of cell differentiation.

The stroma, dense in all specimens, varied in extent. In some instances it was luxuriant and frequently divided the tumour into lobules. In other specimens the tumours were composed almost entirely of epithelial cells.

The epidermis of the outer aspect, that is on the tumour bearing aspect of the auricle, in all cases showed hyperplasia and parakeratosis was frequent (Fig. 7 and 8). In most there was complete loss of hair and in some hyperplastic areas peculiar branching bands of cells somewhat like the fibro-epithelial tumour described by Pinkus (1953) were observed.

A striking feature of the ear lesions was the unchanged state of the epidermis of the inner aspect (Fig. 7 and 9). No tumours were noted on this aspect except where infiltration had occurred from lesions situated on the anterior edge and in the two cases mentioned above where the ear had been amputated. Hyperplasia was the only change observed and this was present in only four of the ears examined. In all other ears the normal 3–4 cell thickness of epidermis was maintained (Fig. 9). This is consistent with solar radiation being an aetiological factor in the pathogenesis of these tumours as the inner apsect of the ear is not exposed to direct sunlight.

All the ulcerated tumours showed considerable inflammation as the result of superimposed infection (Fig. 5). In some tumours in which the keratin cap was still intact, the inflammatory reaction was less marked, while others were free of infection (Fig. 4).

*Muzzle, vulva and eye lesions :* These were all squamous cell epitheliomata and all were markedly infected.

*Connective tissue degeneration.*—A number of sections showed an alteration of the dermal connective tissue analogous to the collagen degeneration observed in the skin of man in those individuals who are prone to develop cancers in which sunlight is presumed to be the cause (Unna, 1896 ; Gillman *et al.*, 1955 ; Mackie and McGovern, 1958). In sheep material stained with haematoxylin and eosin loss of structure of the collagen and associated basophilia was noted. These areas stained light blue with Azure A, pale pink with phosphotungstic acid haematoxylin and yellowish-brown with Van Gieson. Sections stained with Giemsa failed to produce the green reaction commonly seen in human material (McGovern, 1961, personal communication).

Evidence of connective tissue degeneration was seen on the dorsal surface in 11 of the 16 tumour-bearing ears suitable for study, and six of twenty tumour-bearing ears showed the same change on the ventral surface. (The discrepancy in the figures arises from four sections in which the tumour occupied the whole of the dorsal surface.) Ten non-tumour bearing ears were available for comparison One of these showed the change on the dorsal surface, possibly representing an early pre-cancerous stage.

*Metastases.*—In the 28 sheep on which a post-mortem examination was carried out, three cases of metastases to the regional lymph nodes were noted.

The distribution of lesions was as follows :—

| Sheep No. | Site of primary lesion | Site of metastasis |
|---|---|---|
| X 6 | R. ear | R. parotid lymph node which had ulcerated to the surface.<br>R. retropharyngeal lymph node.<br>R. prescapular lymph node. |
| | L. ear (this had been removed, presumed carcinomatous) | L. retropharyngeal lymph node.<br>L. prescapular lymph node.<br>Lung—L. cardiac lobe. |
| X 1 | L. ear | L. prescapular lymph node. |
| X 20 | Both ears | Lymph node. |

If tumour progression (Foulds, 1954) in these animals advanced through benign, malignant and metastasing stages, two factors would have tended to reduce the number of metastases found, namely, early death of the animal from infection or fly strike, and early removal of the lesion by the stockowner. Studies in another group of sheep indicate that occasional tumours of the skin give rise to early metastases. In such cases the effect of the above factors on the incidence of metastases would be less marked.

### DISCUSSION

Papillomata on the ears of sheep in these latitudes is a common finding and, furthermore, it is usual to find a small but fairly constant incidence of malignant neoplasms of the ears and less commonly of other areas exposed to sunlight. Thus when the incidence increased from the expected 0·2 to 1·75 per cent, the possibilities of an increase in the exposure to the carcinogenic agent or the operation of a co-carcinogenic agent were considered.

It appears highly likely that the carcinogenic agent is sunlight in the condition described here. The neoplasia observed closely parallels the occurrence of neoplasms in man in that tumours occur on areas of the body exposed to direct rays of the sun. Furthermore, in the organ particularly exposed, the ear, it is only the exposed surface which is affected except in some instances where the tumour has infiltrated around the edge of the ear to involve the inner aspect. In addition, the condition occurs in animals with non-pigmented skin in a latitude with prolonged solar radiation.

Having established that there was an increased incidence and that the carcinogenic agent was most likely sunlight, it was important to determine whether an increase in the amount of sunlight may have accounted for the higher incidence. Examination of available meteorological records does not support this. The hours of sunlight in the area referred to tend towards the maximum possible. As stated previously, cloud cover is very unlikely to alter the sunlight since it is unusual to get cloudy days, particularly in summer. Cloud leading to rainfall is short-lived as most of the rain falls in rapidly developing thunderstorms. Thus it seems reasonable to dismiss variation in the hours of sunlight as a factor in the cause of the rise in incidence.

The possibility of the influence of a co-carcinogen was considered. The topical application of a co-carcinogenic substance appeared to be highly improbable. The only unusual occurrence that might have contributed to the rise in the incidence of neoplasia was the photosensitisation that had been observed in the flock. Acute photosensitisation with dermatitis and subcutaneous oedema of the ears and face following the ingestion of a number of different plants at certain stages of their growth (*Hypericum perforatum*, *Panicum decompositum* etc.) occurs commonly in Australian sheep, and also occurs in the disease known as " facial eczema " (Cunningham, Hopkirk and Filmer, 1942) in New Zealand following the ingestion by sheep and cattle of grasses infected with the fungus *Pithomyces chararum.*

There are some experimental evidence and field observations to support the idea that photosensitisation may play a role in carcinogenesis. Büngeler (1937) observed a rise in the incidence of epithelioma of the skin in mice treated with chemical carcinogen by producing photosensitisation with eosin and haematoporphyrin. Drabble (1929) noted an association between photosensitisation and epitheliomata of the skin of cattle, and Hore (1961, personal communication) observed unusually high numbers of epitheliomata of exposed parts of the body in sheep in a region where a marked increase in photosensitisation of the "facial eczema" type had occurred.

Thus photosensitisation appears the most likely factor leading to a rise in the incidence of epitheliomata.

There are several pieces of information which are inconsistent with this hypothesis. The first of these is the absence of tumours in the 828 sheep which had been on the property for twelve months. It is certain that some of these were in the susceptible age group of four years and over although the actual number was not known. These would have been subjected both to the initiating agent in similar dosage to the affected sheep throughout their lives and to the promoting agent, photosensitisation, if indeed it is such an agent. One would have expected some cases to appear in these.

Some doubt could be cast on the hypothesis because of the short time elapsing between the operation of the supposed co-carcinogen and the rise in the incidence of neoplasms. Scrutiny of records indicates that although a rise in incidence was noted by the manager during January, February and March 1958, there were 62 cases observed by the author in August 1958 and a further 40 cases in June 1959. As the August 1958 cases would not have survived until June 1959, it is obvious that the latent period was quite long for a substantial proportion of the tumours.

The influence of age on the incidence is of interest. Examination of Table I reveals a lower age limit of about 4 years. Below this there were no cases and above it the incidence was reasonably constant. That there is an influence is consistent with information from other species.

### SUMMARY

A sudden rise in the incidence of squamous cell carcinoma of the ear, muzzle and perineum in a flock of Merino sheep is reported.

Attention is drawn to the occurrence of photosensitisation in the flock preceding the rise in incidence and its possible role as a promoting agent in this species is discussed.

I wish to thank Professor H. R. Carne of this Department for his interest, advice and constructive criticism in the conduct of this project. I am indebted to Burren Station Pty. Ltd. and Mr. M. C. Pearson of Messrs. Goldsbrough Mort & Co. for co-operation, facilities and the sheep used in the detailed study; to Mr. L. E. Whitlock and staff and Mr. R. Jones and staff of this Department for the preparation of sections and photography; to the staff of the Meteorological Bureau, Sydney, and officers of the Soil Conservation Service of N.S.W. for meteorological information; to the Chief Veterinary Officer of Victoria for information regarding the outbreak of "facial eczema" in that State; to Mr. P. Carter, Veterinary Inspector, Coonabarrabran in whose district the flock was situated; to Dr. J. W. Vickery, Botanic Gardens, Sydney, and Mr. D. Jackson, School of Agriculture, University of Sydney, for information regarding pasture species in the area and to Dr. V. J. McGovern, Fairfax Institute of Pathology, Royal Prince Alfred Hospital, Sydney, for helpful advice.

### REFERENCES

ANDERSON, D. E. AND CHAMBERS, D.—(1957) *Misc. Publ. Okla. agric. Exp. Sta.*, No. MP 48, 28.

*Idem*, LUSH, J. L. AND CHAMBERS D.—(1957) *J. Anim. Sci.*, **16**, 739

BEATTI, M.—(1916) *Z. Krebsforsch.*, **15**, 453.

BELISARIO, J. C.—(1959) ' Cancer of the skin '. London (Butterworth & Co.), p. 13.

BLUM, H. F.—(1945) *Physiol. Rev.*, **25**, 483.—(1955) *Rad. Biol.* **2**, 529, cited by Pathak *et al.*, 1959.—(1959) ' Carcinogenesis by ultra-violet light '. Princeton, N.J., U.S.A. (Princeton University Press), cited by Pathak *et al.*, 1959.

BÜNGELER, W.—(1937) *Z. Krebsforsch.*, **46**, 130.

CARTER, P. D.—(1958) *Vet. Insp. N.S.W.*, p. 17.

COOPER, A. G. S.—(1959) *Acta radiol., Stockh.*, Suppl. 188, p. 61.

COTCHIN, E.—(1957) *Vet. Rec.*, **69**, 425.

CUNNINGHAM, I. J., HOPKIRK, C. S. M. AND FILMER, J. F.—(1942) *N.Z. J. Sci. Tech.*, **24**, 185.

DAVIS, C. L. AND SHORTEN, H. L.—(1952) *J. Amer. vet. med. Ass.*, **121**, 20.

DODD, S.—(1923) *J. comp. Path.*, **36**, 231.

DRABBLE, J.—(1929) *Aust. vet. J.*, **5**, 71.

FELDMAN, W. H.—(1931) *Amer. J. Cancer*, **15**, 2044.

FOULDS, L.—(1954) *Cancer Res.*, **14**, 327.

FRENCH, G. T.—(1959) *Aust. vet. J.*, **35**, 474.

GILLMAN, T., PENN, J., BRONKS, D. AND ROUX, M.—(1955) *Arch. Path. (Lab. Med.)*, **59**, 733.

GUILBERT, H. R., WAHID, A., WAGNON, K. A. AND GREGORY, P. W.—(1948) *J. Anim. Sci.*, **7**, 426.

JACKSON, C.—(1936) *Onderstepoort J. vet. Sci.*, **6**, 4.

JOINT BLOWFLY COMMITTEE.—(1943) *Bull. Coun. sci. industr. Res. Aust.*, **174**, 8.

MACKIE, B. S. AND MCGOVERN, V. J.—(1958) *Arch. Derm. Syph., N.Y.*, **78**, 218.

MOLESWORTH, E. H.—(1927) *Med. J. Aust.*, **1**, 877.—(1944) ' An introduction to dermatology '. London (Churchill).

MONLUX, A. W., ANDERSON, W. A. AND DAVIS, C. L.—(1956) *Amer. J. vet. Res.*, **17**, 646.

PATHAK, M. A., DANIELS, F., HOPKINS, C. E. AND FITZPATRICK, T. B.—(1959) *Nature, Lond.*, **183**, 728.

PAUL, W.—(1918) ' The influence of sunlight in the production of cancer of the skin.' London (Lewis).

PINKUS, H.—(1953) *Arch. Derm. Syph., N.Y.*, **67**, 598.

RUSSELL, W. O., WYNNE, E. S., LOQUVAN, G.—(1956) *Cancer*, **9**, 1.

THOMAS, A. D.—(1929) *Rep. vet. Res. S. Afr.* **15**, 659.

TRAUTMAN, A. AND FIEBIGER, J.—(1957) ' Fundamentals of the histology of domestic animals ', translated and revised by R. E. Habel and E. L. Biberstein. Ithaca N.Y. (Comstock Publishing Associates).

UNNA, P. G.—(1896) ' The histopathology of the diseases of the skin ', translated by N. Walker, Edinburgh (Wm. F. Clay).